# Integrated working between residential care homes and primary care: a survey of care homes in England

**DOI:** 10.1186/1471-2318-12-71

**Published:** 2012-11-14

**Authors:** Heather Gage, Angela Dickinson, Christina Victor, Peter Williams, Jerome Cheynel, Sue L Davies, Steve Iliffe, Katherine Froggatt, Wendy Martin, Claire Goodman

**Affiliations:** 1 Department of Economics, University of Surrey, Staghill, Guildford, GU2 7XH, UK; 2 Centre for Research in Primary and Community Care, University of Hertfordshire, Hatfield, AL10 9AB, UK; 3 School of Health Sciences and Social Care, Brunel University, Uxbridge, Middlesex, UB8 3PH, UK; 4 Department of Mathematics, University of Surrey, Staghill, Guildford, GU2 7XH, UK; 5 Department of Primary Care & Population Health, University College London, Royal Free, Campus, Rowland Hill St, London, NW3 2PF, UK; 6 Division of Health Research, Lancaster University, Lancaster, UK

## Abstract

**Background:**

Older people living in care homes in England have complex health needs due to a range of medical conditions, mental health needs and frailty. Despite an increasing policy expectation that professionals should operate in an integrated way across organisational boundaries, there is a lack of understanding between care homes and the National Health Service (NHS) about how the two sectors should work together, meaning that residents can experience a poor "fit" between their needs, and services they can access. This paper describes a survey to establish the current extent of integrated working that exists between care homes and primary and community health and social services.

**Methods:**

A self-completion, online questionnaire was designed by the research team. Items on the different dimensions of integration (funding, administrative, organisational, service delivery, clinical care) were included. The survey was sent to a random sample of residential care homes with more than 25 beds (n = 621) in England in 2009. Responses were analysed using quantitative and qualitative methods.

**Results:**

The survey achieved an overall response rate of 15.8%. Most care homes (78.7%) worked with more than one general practice. Respondents indicated that a mean of 14.1 professionals/ services (other than GPs) had visited the care homes in the last six months (SD 5.11, median 14); a mean of .39 (SD.163) professionals/services per bed. The most frequent services visiting were district nursing, chiropody and community psychiatric nurses. Many (60%) managers considered that they worked with the NHS in an integrated way, including sharing documents, engaging in integrated care planning and joint learning and training. However, some care home managers cited working practices dictated by NHS methods of service delivery and priorities for care, rather than those of the care home or residents, a lack of willingness by NHS professionals to share information, and low levels of respect for the experience and knowledge of care home staff.

**Conclusions:**

Care homes are a hub for a wide range of NHS activity, but this is *ad hoc* with no recognised way to support working together. Integration between care homes and local health services is only really evident at the level of individual working relationships and reflects patterns of collaborative working rather than integration. More integrated working between care homes and primary health services has the potential to improve quality of care in a cost- effective manner, but strategic decisions to create more formal arrangements are required to bring this about. Commissioners of services for older people need to capitalise on good working relationships and address idiosyncratic patterns of provision to care homes.The low response rate is indicative of the difficulty of undertaking research in care homes.

## Background

Long term care for older people in England is provided almost exclusively by the independent sector. The majority of care homes are owned by private organisations or charities, with large chains taking an ever increasing share of the market. In England the Care Quality Commission defines care homes by the type of care residents receive, i.e. care homes with nursing services or those without (sometimes described as residential care). The care of around one half of residents is paid for by the state through their local authority (subject to a means test). The total value of the market in England has been estimated at £22 billion, £16 billion of which is state funded. A small proportion of residents in care homes (with high medical needs) is funded by the National Health Service (NHS) [1-4].

In this paper the term ‘care home’ applies only to care homes that have no on-site nursing provision. The populations they serve predominantly require residential care because of progressive chronic disease and cognitive impairment, resulting in disability and loss of function, and not just for frailty alone [5-7]. These care homes rely on local primary care physicians (general practitioners, GPs) and a variety of community health and social care services for access to medical care and specialist services [1-4]. Despite an increasing expectation that professionals and health and social care organisations should operate in an integrated way across organisational boundaries [8], there is a lack of understanding between care homes and the NHS about how the two sectors should work together [3,9].

Primary and community services spend significant amounts of time providing care for older people resident in care homes. However the service model is often reactive and focused on the individual patient encounter rather than on the population as a whole, distinguished by age and complex range of needs [10-15]. When compared to older people with equivalent needs living in their own homes, those living in care homes have been shown to have less access to care [16-18]. Recognition of unmet health needs, and concerns about unplanned hospital admissions have triggered multiple policy initiatives and interventions specific to care homes [19]. A range of potential solutions have been introduced, including specialist care home teams, and problem-specific workers (e.g. falls prevention) [20,21]. However, reforms have been piecemeal and it is unclear if these new roles have been effective in supporting closer working between primary health care and care homes, or simply increased the extent of service fragmentation.

This paper reports the findings from a survey of care homes without on-site nursing in England. It aimed to establish the current extent of integrated working that exists between care homes and primary and community health and social services. There is not an agreed definition for integrated care. It may refer to formal and strategic partnerships between organisations, that incorporate funding and administrative dimensions [6], and also to looser forms of cooperation, coordination and linkage [22]. This survey adopted a broad definition that focused on activities that seek to ensure that services are coordinated around residents’ needs [23]. It investigated how far integrated care had been achieved through approaches such as joint funding, shared needs assessment and care planning between care homes and local NHS services. It also sought the views of care home managers about the benefits and barriers to integrated working.

## Methods

### Sampling

A national sample of care homes was identified using the online directories held by the Care Quality Commission, the regulator charged with checking whether care services in England meet government standards. Care homes were eligible for inclusion in the survey if: they provided personal care only (no on-site nursing); accommodated only older people (including people with dementia); had 25 places or more (the national average for residential care homes for older people without on-site nursing is 23 with the trend for care homes to increase in size). At the time the study was undertaken (September 2009), there were 2,514 care homes in England that met the inclusion criteria (35% of all residential care homes [24]). Thirty homes were randomly selected to pilot a purpose-designed questionnaire. A systematic random 1 in 4 sample was selected from the remaining homes (n = 621) for the main survey. An email distribution database of care homes was generated from addresses provided in the CQC directory (35%), other internet searches (41%), or phone calls to the home (24%).

### Questionnaire design

A self-completion questionnaire was designed by the research team (Additional file 1), informed by a systematic literature review of integrated working between care homes and primary health care [25], and the different dimensions of integration (funding, administrative, organisational, service delivery, clinical care) that have been identified within and across organisations [8]. Responses were received from four of the 30 (13%) pilot homes (after three reminders), and as a result the survey was shortened, and questions that appeared to have been poorly understood were removed.

The final version (Additional file 1) took between 15 and 20 minutes to complete and consisted of five sections: the primary (GP) and community (list of 26) health care services the care home reported receiving in the previous six months; how the NHS worked with the home, including use of shared documents, joint learning and training, integrated care planning; provision of services for the NHS for which the care home receives specific payment; experiences of integrated working with local health care services, and views about the effects of integration, and barriers to achieving it; characteristics of the care home (region, number of beds, type of registration, number of homes in the organisation, proportion of self- funding residents, staff numbers and qualifications, star (quality) rating of the home at the most recent CQC inspection).

Most questions had a pre-determined response set from which participants selected their answer, although some required numerical input, e.g. number of care home places and staff. In addition, each question had an associated free-text comments box for respondents to add additional information if they wished. Integrated working was defined as ‘close collaboration between professionals and teams (in this case your care home and the NHS) to deliver timely, efficient and high quality care’.

### Distribution of questionnaire

A web-based online version of the questionnaire was set up using Survey Monkey ( http://www.surveymonkey.com/), a feature of which enables anonymous responses, and the mailing of automatic reminders to non-responders. Care home managers were asked, by email, to complete and return the questionnaire within two weeks. To maximise participation, each manager was contacted in advance of the distribution of the survey to explain the purpose of the study and give them warning of when they might expect to receive the questionnaire. After distribution, up to three reminders were sent a week apart to those who had not responded, and managers were invited to contact the research team if they had any questions about the study. As an incentive to participate, return of a completed questionnaire gave entry to a prize draw (for £100 vouchers), and enabled managers to attend one of four national workshops, where the findings would be presented, and they would have the opportunity to network with other care home managers in their area.

### Analysis

Responses from Survey Monkey were imported into SPSS version 17 (SPSS Inc., Chicago, IL, USA) for quantitative analysis. A variable based on the post code of each care home was created to reflect location in an urban (high population density), suburban or rural (low density) area. The characteristics of responding care homes, reported use of primary and community services in the previous six months, and views about integrated working, were analysed descriptively.

Six key indicators of integrated working between care homes and primary / community health services were identified from the survey items: (1) responses to a general question about whether or not any NHS professionals or teams work with the home in an integrated way (Yes / No); (2) the amount of learning and training with NHS colleagues (Weekly / Monthly / Every now and again *vs.* Rarely / Never); (3) use of shared documents (e.g. care plans and notes) with any NHS colleagues (Yes /No); (4) use of integrated care plans with NHS staff, e.g. continence care (All residents and Sometimes *vs.* Never); (5) whether or not the care home received extra payment from the NHS for provision of beds for respite care, palliative / end of life care, continuing care, rehabilitation, day care, to reduce hospital bed use (Yes / No, for each service separately); (6) whether or not the care home reported using more than 1 service (from 26 listed community health and social services) per 3 beds (a standard reached by 64% of homes in the sample) in the previous six months (Yes / No). An overall integration score was derived for each home based on the percentage of the six integration variables for which it had indicated integrated working with the local health service.

Stepwise logistic regression was used to model each integration indicator. Independent variables included in the modelling were: number of beds in the care home; residents per bed (occupancy); number of care homes in organisation; proportion of residents self-funding; whether care home has dementia beds (Yes / No); location in London and SE (*vs.* rest of England); proportion of total staff that are full time (assuming all part time staff are .5FTE); staff: bed ratio; density. Associations between star ratings and each of the integration indicators were explored using an unpaired t test (Pearson’s correlation for overall integration index).

Qualitative data (free text responses to each question) were used to provide contextual information to support the quantitative data. Text was downloaded from the Survey Monkey returns (or entered by hand for hard copy returns) into NVivo8 (QSR International Pty Ltd.) software for analysis. Responses were read and thematically coded. Numbers of responses to individual items varied and comments were often of a general nature relating to more than one question.

### Ethical considerations

A favourable opinion was given by the University of Hertfordshire Ethics Committee.

## Results

### Response rates

Of the 621 homes in the sample, 37 (6%) stated they did not have / did not use email, and were sent the questionnaire by post. The questionnaire was not delivered to 83 of the remaining 584 homes: 49 because the email address was no longer in use, so a paper copy of the questionnaire was mailed instead; 34 (which could not be identified) due to rejection by spam filters. Ninety-three of the 587 care homes receiving the survey completed it (15.8%); 77 / 501 (15.4%) online, 16 / 86 (18.6%) by post. Four homes were subsequently excluded: three had not completed the sections describing characteristic of care home; one was deemed ineligible because it reported only 10 beds (inclusion criteria was > 25 places). This left 89 homes in the analysis. Three homes reporting 22 or 23 beds were retained in the study. Missing responses to individual items occurred frequently and are reported accordingly.

### Characteristics of participating care homes

Most responding care homes were located in urban areas (74%), registered to provide care for people with dementia (60%), and graded good or excellent at their last inspection (83%). Homes reported a mean number of beds of 39, mean occupancy rates of 93%, and around .77 staff (full time equivalent) per resident. The largest proportion of homes were single owner / managed enterprises (31%), with 27% reporting being part of an organisation with over 20 homes (Table 1).

**Table 1:** Characteristics of participating care homes (N = 89)

**Characteristic**		**Responses**	**n**	**%**
**CQC region: London &SE (vs. Rest of England)**		76	28	36.8
**Dementia beds**	**Yes**	75	45	60.0
**Number of care homes in organisation**	**1**	75	23	30.7
	**2-5**		19	25.3
	**6-10**		9	12.0
	**11-20**		4	5.3
	**21-30**		8	10.7
	**> = 31**		12	16.0
**Density**	**Rural**	70	6	8.6
	**Village**		5	7.1
	**Suburban**		7	10.0
	**Urban**		52	74.3
**Number of stars at last inspection**	**0 Poor**	75(mean 2.12)	1	1.3
	**1 Adequate**		12	16.0
	**2 Good**		39	52.0
	**3 Excellent**		23	30.7
			**Mean(Median)**	**SD(Range)**
**Number of beds in home**		75	39.0 (37.0)	10.9 (22–93)
**Residents per bed / occupancy (%)**		65	93.0 (100.0)	11.0 (47–100)
**% of residents who are self funding**		55	42.8 (37.8)	28.7 (0–100)
**% of total staff that are full time**		71	74.7 (86.3)	27.3 (4–100)
**Total full time equivalent staff per bed**		63	.71 (.66)	.20 (.42-1.24)
**Total full time equivalent staff per resident**		59	.77 (.69)	.23 (.42-1.57)

### Primary and community services accessed

All care homes reported receiving services from general practitioners (GPs), and 70 (78.7%) stated they worked with more than 1 practice. Comments revealed that arrangements for GP services can vary. Some homes mentioned scheduled weekly GP clinics, but others described difficulties getting GPs to visit residents in the care home:

‘GPs in this area generally do not like to visit and prefer to diagnose over the phone, which we find unacceptable. We really struggle to get them to visit their patients. It takes months for medication changes to be reflected on repeat prescriptions. Medication reviews only happen at our request apart from one surgery which is very proactive’.

A small number of homes (7, 7.9%) reported that they paid retaining fees to GPs, but comments about this were all negative. Retainers were thought to be unfair:

‘Personally I do not think any care home should pay a retainer, service users have a right to basic medical care and it’s not right that care homes should pay for this. They would get this care free of charge in their own homes and frankly a care home is their home.’

Respondents’ reports indicated that a mean of 14.1 professionals/ services (other than GPs) had visited the care homes (on either an individual or whole home basis) in the last six months (SD 5.11, median 14); a mean (SD) professionals/services per bed of .39 (.163). Almost all homes (> 90%) reported using district nurses (DN) and opticians. Other frequently accessed services were community psychiatric nurses and chiropodists / podiatrists (> 80%).

Between one half and three quarters of homes reported visits from continence nurses, pharmacists, dentists, physiotherapists, occupational therapists, speech and language therapists , dieticians, hearing services and old age psychiatrists (Table 2). However, a consistent theme within the qualitative comments was the difficulty of accessing many specialist services:

**Table 2:** Reported use of community services in the previous 6 months, (either to individual residents or on a full care home basis), ranked by percentage of care homes (N = 89)

**Rank**	**Professional or Service**	**n**	**%**
**1**	District Nurse	84	94.4
**2**	Optician	81	91.0
**3**	Community Psychiatric Nurse	78	87.6
**4**	Chiropody, Podiatry	73	82.0
**5**	Continence service	66	74.2
	Pharmacist	66	74.2
**7**	Dentist	65	73.0
**8**	Physiotherapist	63	70.8
**9**	Occupational therapist	59	66.3
**10**	Speech and Language therapist	58	65.2
	Old age psychiatrist	58	65.2
**12**	Dietician	53	59.6
**13**	Hearing services	51	57.3
**14**	Practice nurse	45	50.6
**15**	Specialist nurse, e.g. older people, diabetes	42	47.2
**16**	Macmillan nurse	39	43.8
**17**	Falls, exercise coordinator	35	39.3
**18**	Consultant geriatrician	33	37.1
**19**	Intermediate care team	30	33.7
**20**	Clinical psychologist	25	28.1
**21**	Community matron	25	28.1
**22**	Hospice team	22	24.7
**23**	Care home support team	18	20.2
**24**	Marie Curie nurse	12	13.5
**25**	Health visitor	9	10.1
**26**	Admirals nurse	4	4.5
**27**	Other	4	4.5
Homes using any palliative service (Macmillan, Marie Curie, or Hospice team)		43	48.3

‘If a Service User requests a visit from any professional we always endeavour to get a home visit at the earliest convenience. Some services attend within days, others can take a long time as they have to wait for a referral from GP etc. This is often the case when trying to get a physiotherapist to call when someone has returned from hospital following hip-replacement surgery.’

‘We have in four years only had one visit from speech and language therapy services, occasionally physiotherapists and occupational therapist services but never from any of the other services with the exception of our chiropodist who we have privately arranged to come every 6–8 weeks.’

### Integration indicators

Many (60%) responding managers considered that they worked with the NHS in an integrated way. Similar proportions reported: accessing at least 1 service for every three beds in the last six months; using shared documents with NHS colleagues; engaging in integrated care planning and joint learning and training (mostly involving district nurses, pharmacists, care home specialist teams, dieticians and community psychiatric services) with NHS. A smaller proportion of homes (37%) reported receiving payment from the NHS for particular services such as respite care (13%), palliative care (11%) and rehabilitation (9%) (Table 3). The mean overall integration score was 54.7%, i.e. on average, homes indicated integrated working with the NHS in just over half of responses to questions on the six key integration variables.

**Table 3:** Integration indicators (N = 89 homes)

**Integration indicator (n,%)**	**Yes**	**No**	**Total**
**Work with NHS professionals /teams in an integrated way**	45 (60.8)	29 (39.2)	74 (100)
**Use > 1 health/social care service in last 6 months per 3 beds**	48 (64.0)	27 (36.0)	75 (100)
**Use shared documents with NHS colleagues**	61 (70.1)	26 (29.9)	87 (100)
**Receive extra payment from NHS for providing specific services**	31 (36.5)	54 (63.5)	85 (100)
**Joint learning and training between care home and NHS**	**Weekly, monthly, every now and again**	**Rarely, never**	
	53 (62.4)	32 (37.6)	85 (100)
**Integrated care planning with NHS colleagues, eg continence care**	**As appropriate, Sometimes**	**Never, Don’t know**	
	47 (59.5)	32 (40.5)	79 (100)

Qualitative data reflect more on the relationship element of care homes working with individual primary / community care staff than integrated working at an organisational level.

‘These responses make it look like we hardly ever work with NHS colleagues whereas we have regular contact with District Nurses and GP with whom we have a good working relationship and liaise closely about individual residents.’

‘We have the best relationships with the GP, district nurses and pharmacist as we work most closely with them’.

Although the survey focused on primary care, many of the care homes indicated that working with secondary care presented major communication difficulties. They reported a lack of mechanisms for structured exchange of information, care planning or follow up of residents transferred to or from hospital:

‘I feel there is a mistrust and poor communication. Transferring a resident to hospital we send all details and then are phoned to ask for them again- poor discharge information to the home which involves possible re-admission to hospital for the resident.’

‘Very poor feedback when a resident returns from hospital and every time a resident is sent to hospital all their notes are sent with them, i.e. medication, abilities, and every time we get numerous calls from the hospital asking for the sent information so not really worth sending it in the first place. This is very frustrating for the home.’

In reality, using shared documentation and assessment tools can mean a range of different things, including the care home completing documentation provided by the NHS. Sharing may be one way- i.e. NHS staff look at or write in care home notes but care home staff do not get reciprocal access to NHS notes. Care home staff indicated that they have skills and knowledge but there were not opportunities to share these with NHS staff:

‘Not sharing per se; more they look at our notes. We then get a copy of any letters produced for Dr's or family, but not access to their notes.’

‘We would like to work more closely with the NHS staff and share our knowledge.’

Regression analysis to explore the care home characteristics associated with integration (each of the six key indicators and the overall integration score) revealed few statistically significant factors. Homes with fewer beds were more likely than larger homes to have used > 1 professional or service per 3 beds in the last 6 months (data not shown). No associations were found between any integration indicator and quality ratings (number of stars) at the last inspection.

### Care home managers’ views about integrated working

Care homes reporting working with the NHS (n = 45, 60.8%, see Table 3) are largely positive about its effects. High proportions of respondents saw the benefits in terms of improving access to services (both therapeutic and preventative), continuity of care and speed of response from the NHS as well as providing opportunities to discuss resident’s care. Even so, over half of this group observed that they felt the NHS was reluctant to share information with care homes (Table 4).

**Table 4:** Views about the effects of integrated working between care homes and NHS (from homes reporting integrated working only (n = 45)

**Integrated working between the** **NHS and my care home has: n (%)**	**N**	**Strongly Agree/ Agree**	**Strongly Disagree/ Disagree**	**Don’t know**
Improved access to preventive care for residents	42	34 (80.9)	8 (19.0)	0
Provided opportunities to discuss resident’s care together	42	31 (73.9)	10 (23.8)	1 (2.4)
Led to greater continuity of service provision	43	30 (69.8)	11 (25.6)	2 (4.7)
Provided a wider range of services for older people	43	32 (74.4)	11 (25.6)	0
Improved the speed of response from primary care	42	31 (73.8)	8 (19.1)	3 (7.1)
Not made residents aware of available services	41	17 (41.5)	22 (53.6)	2 (4.9)
Had no effect on residents quality of life and wellbeing	41	17 (41.5)	22 (53.6)	2 (4.9)
NHS staff are reluctant to share information together	41	17 (41.5)	23 (56.1)	1 (2.4)

Regarding the experiences and perceived barriers to integration, almost one half of responding care homes felt that NHS staff did not provide enough support to care homes, and that care homes did not have enough say when working with the NHS, 43% reported a lack of trust between the NHS and care homes, and over one third stated that they felt they were monitored by the NHS (Table 5).

**Table 5:** Experiences and perceived barriers to integration (N = 89 care homes)

**n (%)**	**N**	**Strongly Agree/ Agree**	**Strongly Disagree/ Disagree**	**Don’t know**
**EXPERIENCES**				
NHS staff provide enough support to help us work effectively	78	40 (51.3)	37 (47.4)	1 (1.3)
NHS staff respect care home staff knowledge and experience	76	30 (39.5)	44 (57.9)	2 (2.6)
Working with NHS staff takes up too much time	78	6 (7.7)	67 (85.9)	5 (6.4)
Sometimes working with the NHS feels like they are monitoring us	76	27 (35.5)	47 (61.8)	2 (2.6)
**BARRIERS**				
It is difficult to know who in the NHS we can ask for information	78	49 (62.8)	28 (35.9)	1 (1.3)
Care home staff don’t have enough say when working with NHS staff	75	42 (56.0)	32 (42.7)	1 (1.3)
Lack of trust between the care home and NHS	77	33 (42.9)	42 (54.5)	2 (2.6)
Staff don’t stay long enough to get to know the NHS staff	71	9 (12.7)	62 (87.3)	0
It is important to have a named person we can contact	77	74 (96.1)	3 (3.9)	0
Staff don’t stay long enough to get involved in training with NHS staff	71	6 (8.5)	63 (88.7)	2 (2.8)
We cannot work together well because of different priorities	75	16 (21.3)	57 (76.0)	2 (2.7)

Open text comments indicated that some care homes perceived differences in working cultures and priorities, and a lack of understanding of the role of care homes, as contributing to poor working relationships with NHS staff. Care home staff felt strongly that their knowledge of residents should be listened to by NHS staff and respected:

‘Some NHS staff do not understand the workings of a care home and that it is in fact "home" to the residents.’

‘We are not qualified nurses but do know our residents better than a stranger who may see them for 10 minutes. ‘

## Discussion

The national survey found that residential care homes with no on-site nursing are a hub for a wide range of NHS activity with up to 26 different services identified. On average, homes reported accessing between 14 and 15 different professionals or services in the six months before the survey, with the highest proportion of homes reporting links with DNs, opticians, chiropodists/podiatrists, community psychiatric nurses and continence services. However there was no single recognised way in which homes and primary care services work together. Arrangements continue to be *ad hoc*, and largely dependent on individual relationships between care home staff and NHS professionals.

Three levels of collaboration have been identified, ranging from linkage, through co- ordination to full integration [25]. The survey findings suggest that most collaboration between care homes and primary care services are linkages, fostered by good working relationships between care home staff and NHS professionals [26]. These were perceived to be more important than particular systems or processes, but were person-specific, and vulnerable to change. There was evidence of some coordination at a clinical level, (e.g. shared care planning and joint training). Also, some care homes reported holding contracts with the NHS for provision of extra services, such as respite care, but this did not appear to affect working practices or be associated with more integrated patterns of working. Collaboration thus appeared to be largely at the lower level of linkage and co-ordination, determined by the powers of actors who are working on the front- line of service delivery, and limited by operational factors. Care homes reported that working practices were dictated by NHS methods of service delivery and priorities for care, rather than those of the care home or residents. Moreover, there was often a lack of willingness by NHS professionals to share information, and perceived low levels of trust and respect for the experience and knowledge of care home staff.

Given the frailty and complex needs of the care home population, more integrated working between care homes and primary health services has been promoted as a cost-effective means of improving the quality of care [23]. With increasing financial pressures on health and social care resources [27], the focus on integrated care processes as a mechanism to improve co-ordination, efficiency and value for money of patient care is likely to increase [28-30]. Integrated working has been described as a dynamic process, developing over time, and requiring trusted leaders who use organisational systems and processes to work together to fulfill shared goals [31]. The survey identified many examples of positive working relationships between health care and care home staff, but few examples of systems of working that were recognised as supporting truly integrated working. Future mechanisms for commissioning integrated care services will be through Commissioning Care Groups, supported by the NHS Commissioning Board, and workable frameworks to support decision making about contracting and procurement of services will need to be agreed [28].

### Strengths and Limitations

The strengths of the study are that the questionnaire was carefully prepared and piloted, the sampling was systematic, reminders and other means were used to try and boost the response rate, and the findings were rigorously analysed using a mix of quantitative and qualitative methods. The results provide up-to-date information on a currently important issue where little evidence already existed.

Survey work in care homes is difficult to conduct [32,33], and a major limitation of the study is the poor response rate (16%). The questionnaire was shortened considerably after piloting, but it was not set up with required fields, or to block inconsistent answers, missing items and non-logical responses, and this limited the analysis to some extent. Although homes were invited to ask for a paper version of the survey, (and a small number did), the online method of data collection may have been inappropriate for a sector that anecdotally is seen as having limited online capability. Surveys of physicians have shown lower response rates from online compared to other methods [34].

Problems arose within the survey regarding the interpretation of the concept of integration. A definition of integrated working was provided to respondents (close collaboration between….your care home and the NHS), but this did not provide sufficient information to enable care home managers to distinguish between loose linkages, coordinated care and formal collaborative arrangements, or to relate sufficiently to the concept of integration. Significant proportions (30-60%) of home managers that stated that they did not work with the NHS in an integrated way reported that they did engage in activities that were used in the study as indicators of integration (joint learning and training, shared documents, integrated care planning, provision of remunerated services) used in the study. Such inconsistencies might have been avoided if explanation of the nuances surrounding the concept of integration had been made clearer to respondents. The finding that no care home characteristics were associated with reporting of any of the integration indicators used in the study may further reflect lack of understanding of the practical manifestations of integrated working.

The study only aimed to survey homes with 25 or more beds due to the logistical difficulties of covering the large number of smaller residential facilities. The nature and extent of collaboration between care homes and the local NHS may differ amongst smaller homes, and the views of their managers about the benefits and barriers may not accord with those of managers of the larger homes. The survey instrument used in this study was also completed by 102 homes in a major chain. Homes in the chain (in line with recent trends for increasing care home size) were significantly larger than those in the national survey reported here (mean 55.3 *vs.* 39.0 beds, Students t test p < .001), and were significantly more likely to provide extra remunerated services for the NHS than homes in the national sample (58% vs. 36%, chi square p = .002). There were few other differences between the samples regarding integration indicators or managers’ views (full data not shown).

## Conclusions

This paper provides contemporary evidence from a national survey of the state of working between care homes and primary health care services, as a basis for policy-making and service planning, and as a benchmark against which future progress may be measured. In line with other recent work [35], the findings suggest that integration between care homes and local health services is mainly evident at the level of individual working relationships and reflects patterns of collaborative working rather than integration. Contrary to expectations the survey did not find a pattern of increasing activity and collaboration when compared with an earlier survey [18]. Existing recommendations point to the need for care homes to receive more support from local primary health care services [12], and organising this through integrated care mechanisms has the potential to generate maximum enhancements to service quality for residents, and efficiency gains for the delivery organisations [8]. Commissioners of services for older people in need of long-term care should capitalise on existing examples of good working relationships between care homes and the NHS, and address idiosyncratic patterns of provision, including lack of clarity about responsibilities and budgetary constraints between the two sectors. A national survey of NHS services to care homes confirms the findings of this study, finding inadequate understanding of what effective service provision looks like [36]. Further empirical research is therefore required to explore in more detail which methods, processes, and models of integrated care are most effective in terms of improving access, health outcomes and quality of care for the care home population [3]. Research should include service-user perspectives which are central to the delivery of integrated care [23].

## Competing interests

The authors declared that they have no competing interest.

## Authors' contributions

All authors contributed to the design of the questionnaire. SLD constructed the sample, distributed the questionnaire, and, with assistance from JC, organised the data. PW, JC, HG undertook the quantitative and statistical analysis. AD, CV undertook the qualitative analysis. HG, AD, CV drafted the manuscript. KF, SLD, SI and CG edited the manuscript. CG conceived of the study, and with HG, AD, CV, SI, KF and WM contributed to the study design and funding application. All authors read and approved the final manuscript.

## Pre-publication history

The pre-publication history for this paper can be accessed here:

http://www.biomedcentral.com/1471-2318/12/71/prepub

## Supplementary Material

Additional file 1 Survey questions.pdf, 287K. Click here for file
