# Supplementary material for: Integrated working between residential care homes and primary care: a survey of care homes in England
Source: BMC Geriatr. 2012 Nov 14;12:71. doi: 10.1186/1471-2318-12-71 (PMC3534387; doi:10.1186/1471-2318-12-71)
Supplement: Additional file 1 — Survey questions.pdf, 287K. [file 1471-2318-12-71-S1.pdf]

APPROACH SURVEY

WELCOME TO THE APPROACH National Care Home Survey and opportunity to win PRIZE of £100 M&S vouchers.

We are inviting you to participate in this study by completing an on-line questionnaire, which should take approximately 15-20 minutes to complete.

There are five sections:

Section A: Primary health care services
Section B: How you work with the NHS
Section C: Experiences of integrated working with the NHS
Section D: Information about your care home
Section E: Care home staff

We have tried to design the questionnaire to be as easy as possible to complete. Most of the questions just ask you to tick a box. Comment boxes have been provided should you wish to add further information to your answer.

All completed questionnaires are anonymous and will be treated with the strictest confidence. Any information given through which your home could be identified will be removed or changed.

Your views are important and we hope that you will take the time to complete the questionnaire. If you would like to discuss any aspect of the study, please do contact me.

We really appreciate your help with this study, and to show our appreciation there is an opportunity to be entered into a prize draw to win £100 of vouchers for Marks and Spencers.

Sue Davies
Approach Research Fellow
s.l.davies@herts.ac.uk
Tel: 01707 289375

APPROACH SURVEY

SECTION A: The following questions are looking at the primary health services your care home receives and how the NHS works with you.

1. Do you CURRENTLY have more than one GP practice working with the care home?

- Yes
- No

If yes, how many practices work with your care home?

2. If you pay a retainer to your main GP practice, please tick the amount below

- No retainer paid
- Less than £1,000 per year
- £1000-£4,999
- £5,000-£9,999
- £10,000-£14,999
- £15,000-£19,999
- £20,000-£24,999
- £25,000 or over
- Don't know

Any comments

3. Do you pay more than one GP practice a retainer?

- Yes
- No

Comments

4. What services do you get from the GP practice you have most contact with?

	Always	Occasionally	Never	Don't know
Visits individual residents when they are unwell				
Telephone advice when residents are unwell				
Reviews the medication of ALL residents				
Reviews the medication of INDIVIDUAL residents				
Refers residents to specialist services (e.g. geriatrician)				
Provides advice on keeping ALL residents healthy				
Provides advice on keeping INDIVIDUAL residents healthy				
Offers training and advice to staff				

Any comments

5. Do any GPs undertake regular (at LEAST fortnightly) 'clinics' located in the care home?

- Yes
- No

Please give details

6. Are residents able to self-refer to the GP 'clinic' in the care home?

- Yes
- No
- Not applicable

Any comments/ further information

7. Do other health care professionals hold drop in type surgeries in the care home (e.g. nurses, chiropodists, physios)?

- Yes
- No

If YES, Please give details

8. Which of the following health and social care professionals have visited the care home in the LAST SIX MONTHS? Please tick all that apply

	We have NOT received this service in the LAST SIX MONTHS	On a resident by resident basis ONLY	Provides a designated service to the whole care home	We pay for this service	We support residents to attend services OUTSIDE the care home.
District Nurse					
Pharmacist					
Chiropody/podiatry					
Practice Nurse					
Community Matron					
Older people's nurse specialist (e.g. Tissue viability/diabetes)					
Health visitor					
Care home support team					
Community psychiatric nurse/mental health team					
Old age psychiatrist					
Clinical psychologist					
Dietician					
Continence Team					
Dentist					
Optician					
Hearing aid services (audiometry)					
Hospice Team					
Marie Curie service					
Macmillan nurse/specialist palliative care nurse					
Consultant geriatrician					
Speech and language therapist					
Occupational therapist					
Physiotherapist					

APPROACH SURVEY

Intermediate care team	(	(	(	(	(
Falls prevention activities/Exercise co-ordinator	(	(	(	(	(
Admiral Nurse	(	(	(	(	(
Other (please state below)	(	(	(	(	(

Any comments

9. Of those services that have visited the care home in the last SIX MONTHS, please tick all that apply

	NOT APPLICABLE	The frequency of visits depends on individual residents	We have a named contact for this service	We meet to discuss working together for particular residents	We meet to discuss working together for ALL residents
GP	e	e	e	e	e
District Nurse	e	e	e	e	e
Pharmacist	e	e	e	e	e
Chiropody/podiatry	e	e	e	e	e
Practice Nurse	e	e	e	e	e
Community Matron	e	e	e	e	e
Older people's nurse specialist (e.g. tissue viability/diabetes)	e	e	e	e	e
Health visitor	e	e	e	e	e
Dietician	e	e	e	e	e
Care home support team	e	e	e	e	e
Community psychiatric nurse/mental health team	e	e	e	e	e
Old age psychiatrist	e	e	e	e	e
Clinical psychologist	e	e	e	e	e
Continence services	e	e	e	e	e
Dentist	e	e	e	e	e
Optician	e	e	e	e	e
Hearing aid services (audiometry)	e	e	e	e	e
Hospice Team	e	e	e	e	e
Marie Curie service	e	e	e	e	e
Macmillan nurse/specialist palliative care nurse	e	e	e	e	e
Consultant geriatrician	e	e	e	e	e
Speech and language therapist	e	e	e	e	e
Occupational therapist	e	e	e	e	e
Physiotherapist	e	e	e	e	e
Intermediate care team	e	e	e	e	e
Falls prevention activities/Exercise co-ordinator	e	e	e	e	e
Admiral Nurse	e	e	e	e	e
Other (please state below)	e	e	e	e	e

Any comments

10. Do you use shared documents with any of your NHS colleagues mentioned above, e.g. care plans and notes.

- Yes (Please go to question 11)
- No (Go to question 12)
- Don't know

11. If yes, which colleagues do you share notes/care plans etc with? (Please list)

- GP
- District Nurse
- Pharmacist
- Older people's nurse specialist (e.g. diabetes, tissue viability)
- Community psychiatric nurse/mental health team
- Care Home Support Team
- Intermediate care team
- Dietician
- Geriatrician
- Macmillan Nurse/specialist palliative care nurse
- Other (please specify below)

Other (please specify)

12. We do learning and training together with NHS colleagues.

- Weekly
- Monthly
- Every now and again
- Rarely
- Never (Go to question 14)

Comments

13. Which NHS staff do you do learning and training with? (Please list)

- GP
- District Nurse
- Pharmacist
- Older people's nurse specialist (e.g. diabetes, tissue viability)
- Community psychiatric nurse/mental health team
- Care Home Support Team
- Intermediate care team
- Dietician
- Geriatrician
- Macmillan Nurse/specialist palliative care nurse
- Other (please specify below)

Other (please specify)

14. Please tick which of the following you use when working with the NHS

	Not sure what this is	Use this for all our residents when appropriate	Use sometimes	Never use it
Integrated care plans with NHS staff e.g. continence care				
Assessment tools/shared decision support tools e.g. MUST (nutrition screening tool)				
Protocols for addressing behaviour				
Dementia assessment tool				
Essence of Care				
Advance care plans for end of life care				
Gold standard framework (GSF)				
Liverpool care pathway				
Single assessment process				
Medication review policy				
Infection control protocols				

Any comments

SHARED FINANCIAL ARRANGEMENTS

By NHS we mean GP services as well as other professionals such as district nurses, occupational and physiotherapy etc.

15. Please could you indicate if you receive extra PAYMENT FROM THE NHS (in addition to care home fees) to provide any of the following

	Don't know	Paid for individual residents	Paid to keep beds available in the care home	Arrangement has been in place for less than 6 months	Care home does not provide this service
NHS funded respite care beds					
NHS funded palliative end of life beds					
NHS funded continuing care					
NHS funded rehabilitation					
NHS funded beds to reduce hospital bed use					
NHS funded day care					

Any comments

16. Please indicate how the following services are paid for in your care home?

	NHS	Local authority/social care	Mixed - some residents pay others paid by LA or NHS	Private (individual resident pays)	Part of care home organisation
Podiatry/chiropody					
Physiotherapy					
Occupational therapy					
Speech and language therapy service					
Palliative care support					
Care home support team					

Any comments

17. Of the NHS services you work with, who do you have the best working relationships with?

- GP
- District Nurse
- Pharmacist
- Older people's nurse specialist (e.g. diabetes, tissue viability)
- Community psychiatric nurse/mental health team
- Care Home Support Team
- Intermediate care team
- Dietitian
- Geriatrician
- Macmillan Nurse/specialist palliative care nurse
- Other (please specify below)

Other (please specify)

18. What would you like primary health care services to provide for your care home that you are not currently getting?

19. What would help you to work more closely with the NHS?

APPROACH SURVEY

Integrated working can be defined as close collaboration between professionals and teams (in this case your care home and the NHS) to deliver timely, efficient and high quality care.

The following questions look at the ways that integrated working with the NHS may have affected your care home.

By NHS we mean services such as GP, district nursing, occupational and physiotherapy etc.

IF THIS DOES NOT APPLY TO YOUR CARE HOME, PLEASE GO TO QUESTION 23.

20. Are there any NHS professionals or teams that work with the care home in an integrated way? (current or previous)

- Yes (Please give details below)
- No (Go to question 23)

Please give further details

21. Integrated working (as described above) between the NHS and my care home has:

	Strongly agree	Agree	Disagree	Strongly disagree	Don't know
Not made the residents more aware of available services					
Provided a wider range of choice of services to older people					
Improved access to preventative care for residents					
Had no effect on resident's quality of life and well-being					
Improved the speed of response from primary health care					

Any comments

22. Integrated working between the NHS and my care home has:

	Strongly agree	Agree	Disagree	Strongly disagree	Don't know
Provided opportunities to discuss resident's care together					
Led to greater continuity of service provision					
NHS staff are reluctant to share information with us					

Any comments

23. To what extent do you agree with each of these statements about working relationships between the NHS and your care home?

	Strongly agree	Agree	Disagree	Strongly disagree	Don't know
NHS staff provide enough support to help us work effectively					
NHS staff respect care home staff knowledge and experience					
Working with NHS staff takes up too much time					
Sometimes working with the NHS feels like they're monitoring us					

Any comments

24. To what extent do you agree that the following factors present BARRIERS to integrated working with the NHS for your care home? Please tick one response per statement

	Strongly agree	Agree	Disagree	Strongly disagree	Don't know	Not applicable
It's difficult to know who in the NHS we can ask for advice/information	€	€	€	€	€	€
Care home staff don't have enough say when working with NHS staff	€	€	€	€	€	€
There is a lack of trust between the care home and the NHS	€	€	€	€	€	€
Staff don't stay long enough to get to know NHS staff	€	€	€	€	€	€
It is important to have a named person we can contact	€	€	€	€	€	€
Staff don't stay long enough to get involved in training with NHS staff	€	€	€	€	€	€
We cannot work together well because of different priorities	€	€	€	€	€	€

Any comments

5

6

25. Are there any other BARRIERS that affect integrated working with your care home. Please state

5

6

APPROACH SURVEY

The following section asks you about your care home:-

26. Which Care Quality Commission region is your care home based in?

- East Midlands
- Eastern
- London
- North East
- North West
- South East
- South West
- West Midlands
- Yorkshire and Humberside

Please insert the first part of your postcode

27. How many beds does your care home have?

28. Which of the following categories of registration does your home provide beds under? Please tick all that apply.

- Non-specialised
- Dementia care
- Intermediate care
- Hospice
- Respite
- Step down
- Step up
- NHS Beds
- Elderly frail
- Other (please specify)

29. Please enter the number of beds you have for these categories:

Non-specialised beds

Dementia care

Intermediate care

Hospice

Respite

Step down

Step up

NHS beds

Elderly frail

Other

30. Care home ownership type

- Local authority
- Voluntary/Charity/Not for profit
- Private/For profit

31. How many care homes are there in the organisation?

- 1
- 2-5
- 6-10
- 11-20
- 21-30
- 31+

Any comments

32. Of the total current residents, how many are in the following funding categories? Please insert a number

Wholly self funding (or family) funded

Wholly Local Authority funded

Mixed self or family and Local Authority funded

NHS funded

Wholly funded by a charity

APPROACH SURVEY

33. Please indicate the outcome of most recent inspection for your care home (Care quality commission) Please select one

- ***
- **
- *
- Zero stars
- Not known

APPROACH SURVEY

About the current staffing in the care home

34. How many full time staff do you have who work with you in the care home (including night staff)?
Please insert the number

Managers

Care staff NVQ2 and above or equivalent

Care staff without NVQ2

Activity co-ordinator

Students on placement

35. How many part time staff do you have? Please insert the number

Managers

Care staff NVQ2 and above or equivalent

Care staff without NVQ2

Activity coordinator

Students on placement

36. Have 50% of your staff achieved NVQ2 or above?

Yes

No

Any comments

THANK YOU VERY MUCH FOR TAKING THE TIME TO COMPLETE THIS SURVEY.

YOUR VIEWS ARE REALLY IMPORTANT.

IF YOU WOULD LIKE TO BE ENTERED INTO THE PRIZE DRAW FOR A £50 MARKS AND SPENCER'S VOUCHER PLEASE GIVE YOUR CONTACT DETAILS BELOW. YOUR DETAILS WILL NOT BE USED FOR ANY OTHER REASON AND WILL BE REMOVED BEFORE ANY ANALYSIS OF THE SURVEY INFORMATION.

37. Please give your name, email / phone number for the draw:
